# Catheter-directed therapy with the FlowTriever system for intermediate-high and high-risk pulmonary embolism: a single-centre experience

**DOI:** 10.1007/s12471-024-01916-1

**Published:** 2024-12-10

**Authors:** Einar A. Hart, Paul Eenhoorn, Mathilde Nijkeuter, Dieuwertje Ruigrok, Joris J. van der Heijden, Michiel Voskuil, Tommy K. K. Liu, Jan Willem Balder, Tim P. van de Hoef, Pim van der Harst, Adriaan O. Kraaijeveld, Michael G. Dickinson

**Affiliations:** 1 Department of Cardiology, University Medical Centre Utrecht, Utrecht, The Netherlands; 2 Department of Intensive Care, University Medical Centre Utrecht, Utrecht, The Netherlands; 3 Department of Internal Medicine and Dermatology, University Medical Centre Utrecht, Utrecht, The Netherlands; 4 Department of Pulmonology, University Medical Centre Utrecht, Utrecht, The Netherlands

**Keywords:** Pulmonary embolism, Mechanical thrombosuction, FlowTriever, Systemic thrombolysis

## Abstract

**Background:**

Pulmonary embolism is an important cause of preventable mortality. Treatment strategies depend on risk stratification. High-risk patients, and some intermediate-high-risk patients, require urgent reperfusion therapy. Systemic thrombolysis (ST) is the effective first-choice treatment in these patients; however, the high risk of bleeding complications is a major drawback. In this single-centre retrospective cohort study, we report our experience with the FlowTriever thrombosuction system as an alternative or adjunct to ST in intermediate-high and high-risk pulmonary embolism.

**Methods:**

Demographic and clinical data of all patients treated with the FlowTriever system from December 2021 until March 2024 were retrieved from the electronic medical records. Primary outcomes were technical success rate, 30-day all-cause mortality and major bleeding.

**Results:**

Twenty-one patients were treated with the FlowTriever system, 14 of whom were considered high risk. The technical success rate was 100%. Thirty-day all-cause mortality was 29% and major bleeding was recorded in 5 patients (24%), of which one bleeding event was related to the FlowTriever procedure. A significant reduction was seen in mean pulmonary arterial pressure and right ventricular end-diastolic dimension.

**Conclusion:**

In intermediate-high and high-risk pulmonary embolism patients with ST treatment failure or a contraindication for ST, the FlowTriever thrombosuction system seems to be a minimally invasive alternative treatment modality with low complication rates.

**Supplementary Information:**

The online version of this article (10.1007/s12471-024-01916-1) contains supplementary material, which is available to authorized users.

## What’s new?

- Patients with intermediate high-risk or high-risk pulmonary embolism with a contraindication for systemic thrombolysis, or when thrombolysis has failed, pose a challenge for treating physicians.
- This study reports on the use of the FlowTriever system in the Netherlands, a device for mechanical thrombosuction which removes the need for thrombolysis.
- The FlowTriever system is a safe alternative for, or adjunct to, systemic thrombolysis with a high technical success rate and low procedural complications.

## Background

**Fig. 1:**
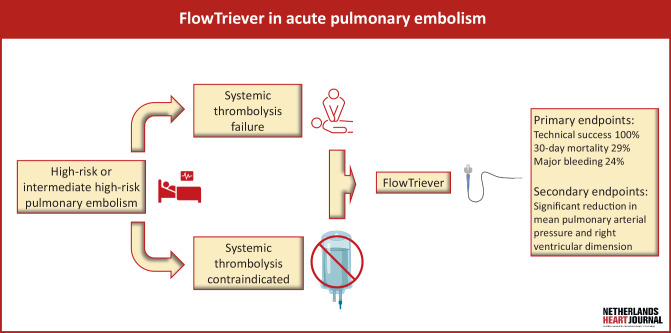
Infographic: FlowTriever in acute pulmonary embolism

Pulmonary embolism (PE) is an important cause of preventable mortality, with an increasing incidence driven by an ageing population and improved detection [1, 2]. Risk factors include major surgery, immobilisation, malignancy, pregnancy and inflammatory states [3]. PE is classified as low risk in the absence of simplified Pulmonary Embolism Severity Index (sPESI) criteria, intermediate-low risk with signs of right ventricular (RV) dysfunction or elevated cardiac biomarkers, intermediate-high risk when both RV dysfunction and elevated biomarkers are present and high risk when presenting with haemodynamic instability. Guidelines recommend oral or parenteral anticoagulation for low- and intermediate-risk PE, additional close monitoring for intermediate-high-risk PE and urgent reperfusion therapy for high-risk PE [4]. In rare cases, intermediate-high-risk PE will deteriorate and require rescue reperfusion therapy to prevent acute RV failure and mortality [5]. In the largest analysis of high-risk PE patients to date, a mortality rate of 20.6% was reported [6]. The first-choice reperfusion therapy in this patient category is systemic thrombolysis (ST) with recombinant tissue-plasminogen activator. Although ST is effective in lysing clots and subsequently decreasing RV afterload with ameliorating pulmonary perfusion, its major drawback is the occurrence of major bleeding complications in up to 42% of patients [6, 7]. Depending on centre experience, catheter-directed therapy and surgical embolectomy can serve as adjuncts when ST fails or as alternatives in patients predisposed to a high risk of bleeding. The most well-studied catheter-directed device combines catheter-directed thrombolysis with ultrasound fragmentation. Meta-analysis showed relatively few major bleeding complications (7%) and low mortality (3.6%) in high-risk PE [8]. However, we previously observed higher major bleeding rates (34%) and mortality (49%) in a real-world cohort of high-risk patients, 70% of whom had contraindications for ST [9]. More recently, the use of thrombosuction systems has emerged, with the advantages of directly restoring pulmonary perfusion by clot removal and avoiding (additional) thrombolytic therapy. One of these systems is the FlowTriever device (Inari Medical, Irvine, CA, USA), a catheter-directed mechanical thrombectomy device using large-bore venous access. The system removes the need for local or systemic thrombolysis. The FlowTriever device was introduced in the Netherlands in 2021; however the outcomes of the FlowTriever system in the Netherlands have not yet been described. This cohort analysis describes our first experience with the FlowTriever system for intermediate-high and high-risk PE patients (Fig. 1).

## Methods

### Patient selection

**Fig. 2:**
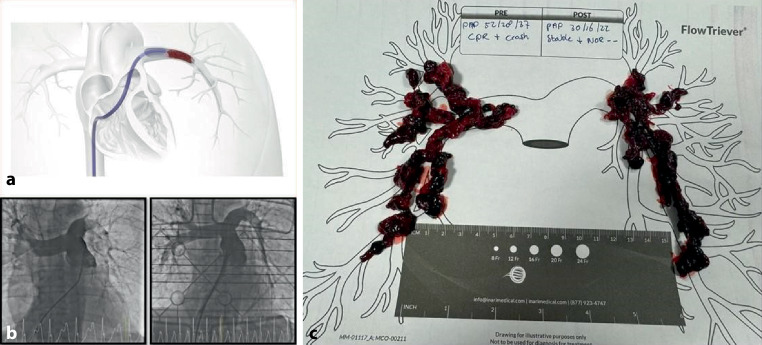
FlowTriever catheter located in left pulmonary artery (**a**), pulmonary angiogram (**b**) showing resolution of thrombus load after the FlowTriever procedure, thrombus yield (**c**). (Printed with permission from Inari)

This cohort study was conducted in the University Medical Centre in Utrecht, the Netherlands. All patients treated with the FlowTriever system (Fig. 2) from December 2021 until March 2024 were included. PE was diagnosed using computed tomography pulmonary angiography (CTPA) or fluoroscopic pulmonary angiography in the catheterisation laboratory. Risk stratification into intermediate-high or high-risk PE was performed according to European Society of Cardiology/European Respiratory Society guideline definitions [4]. Treatment decisions were made by a multidisciplinary PE response team consisting of a pulmonologist, interventional cardiologist, intensive care physician and internal medicine physician. Intermediate-high and high-risk PE were treated with thrombosuction when ST was contraindicated because of a high bleeding risk and/or when ST did not ensure rapid clinical improvement (failure). The definition for ST failure was largely based on clinical judgement and included 1) no improvement in the clinical profile after several hours following ST initiation, or 2) deterioration after ST where death seemed imminent. Risk factors for ST were based on European Society of Cardiology/European Respiratory Society guidelines [4]. Absolute contraindications for ST included a recent (< 6 months) history of stroke, central nervous system neoplasm, major trauma (< 3 weeks), surgery (< 3 weeks), head injury (< 3 weeks), bleeding diathesis or active bleeding. Relative contraindications included recent transient ischaemic attack (< 6 months), oral anticoagulation, traumatic resuscitation, pregnancy and advanced liver disease. There were no exclusion criteria. Demographic and clinical data were retrieved from electronic medical records. Primary outcomes were technical success rate (defined by successful delivery of the device, operation of the device and removal of the device as described by others [10, 11]), all-cause 30-day mortality and major bleeding complications. Major bleeding complications were classified according to International Society on Thrombosis and Haemostasis criteria, defined as (1) fatal bleeding and/or (2) symptomatic bleeding in a critical area or organ, such as intracranial, intraspinal, intraocular, retroperitoneal, intra-articular or pericardial, or intramuscular with compartment syndrome and/or (3) bleeding causing a fall in the haemoglobin level of 20 g l^−1^ (1.24 mmol l^−1^) or more, or leading to transfusion of two or more units of whole blood or red cells. [12]. Secondary outcomes were reduction in mean pulmonary artery pressure (mPAP) and reduction in RV end-diastolic (basal) dimensions (RVEDD).

### The procedure

Echo-guided venous access is mainly obtained via the femoral vein. In this series, one patient was treated via the internal jugular vein, since femoral access had already been used for veno-arterial extracorporeal membrane oxygenation (VA-ECMO). After securing access with a 7 Fr. sheath, the patient is heparinised with a target activating clotting time (ACT) of 250–300 s. Then a balloon-tipped catheter (or Swan Ganz) is used to safely pass the tricuspid valve apparatus and pulmonary valve up into the pulmonary trunk. Ideally, baseline haemodynamic measurements are taken at this point. Using an exchange wire, the balloon-tipped catheter is exchanged for a pigtail catheter for a pulmonary angiogram. After switching to a multipurpose catheter, the catheter is then safely positioned deep in the vascular tree bed, either with the use of a hydrophilic coated wire or a coronary wire. Through the catheter, an extra-long stiff wire (e.g. Amplatz Super Stiff with a 1 cm floppy J tip, Boston Scientific) is positioned well distal of the targeted clots, maintaining this position throughout the procedure. The venous access is then predilated up to the 24 Fr. large-bore sheath (Inari Medical). Through the sheath, an over-the-wire aspiration catheter (triever24, 20 or 16, Inari Medical) is guided up into the pulmonary clots. In this position the inner dilator is then removed and the large-bore syringe is attached to the catheter’s flush port creating a vacuum for thrombus aspiration through the catheter. While maintaining vacuum on the system, multiple aspirations are then performed with repositioning of the catheter if necessary. To further engage or disrupt clots, nitinol discs (FlowTriever catheter) or a basket (FlowTriever2 catheter) can be backloaded through the aspiration catheter, while maintaining vacuum on the entire system. Also, using a mother-child technique, the Triever Curved catheters (16 or 20 Fr, Inari Medical) can be used for curved anatomy. In order to reduce blood loss during the procedure, the FlowSaver system (Inari Medical) is used to filter aspirated blood from all clots, allowing for direct reinfusion of filtered blood through the side port of the sheath using a 50 cc syringe. This procedure is then repeated until a satisfactory result is reached in both lungs, followed by haemodynamic evaluation post procedure. The femoral vein can be closed either using a suture-based closure device (e.g. Perclose Prostyle, Abbott; Flowstatis suture, Inari Medical) or a common ‘*figure of 8’* suture.

### Statistics

Variables were reported as median (interquartile range) or mean (standard deviation). Survival was displayed using a Kaplan-Meier curve. The Wilcoxon signed-rank test was used for comparison between related groups. Statistical analyses were performed with IBM SPSS Statistics, version 29.0.1 (IBM Corp.).

## Results

During the study period, 21 patients were treated using the FlowTriever system, 14 of whom were classified as high risk and 7 as intermediate-high risk. Ten patients (48%) presented with circulatory arrest. Patient and interventional characteristics are shown in Tab. 1. The procedural characteristics and individual patient characteristics and outcomes are shown in the Electronic Supplementary Material.

**Table 1:** Patient and interventional characteristics

	Intermediate-high risk (*n* = 7)	High risk (*n* = 14)	Total (*n* = 21)
Age, years	58 (39–68)	56 (44–66)	56 (42–66)
Female	6 (86%)	7 (54%)	13 (62%)
BMI, kg/m^2^	26.9 (23.2–29.0)	26.0 (21.9–31.3)	26.9 (22.7–30.0)
Hypertension	0	2	2
COPD	1	0	1
Coronary artery disease	0	0	0
Stroke/TIA	0	1	1
Venous thromboembolism*	1	6	7
Active malignancy	1	2	3
Absolute contraindication for ST	7	8	15
Relative contraindication for ST	0	3	3
ST failure	0	9	9
ST contraindicated	7	5	12
Circulatory arrest	0	10	10
VA-ECMO	0	6	6
Mechanical ventilation	2	12	14
Systolic BP (*n* = 13), mm Hg	128 (108–140)	109 (90–120)	110 (94–132)
Diastolic BP (*n* = 13), mm Hg	80 (77–88)	65 (58–78)	76 (62–81)
Heart rate (*n* = 15), bpm	106 (99–116)	90 (86–120)	106 (87–119)
Serum lactate (*n* = 14), mmol/l	1.4 (n/a)**	5.6 (2.4–9.4)	5.1 (1.4–9.4)
Haemoglobin (*n* = 18), mmol/l	5.7 (5.0–8.5)	7.3 (6.2–8.7)	6.6 (5.7–8.4)

* Including previous pulmonary embolism

** *n* = 3 and includes one patient with pulmonary embolism and septic shock with a lactate of 12.6 mmol/l. This patient was considered intermediate-high risk as no clear right ventricular dysfunction was observed; hence the elevated lactate was considered to be a reflection of distributive shock. Data are displayed as number (%) or median (interquartile range)

*BMI* body mass index, *BP* blood pressure, *BPM* beats per minute, *COPD* chronic obstructive pulmonary disease, *ST* systemic thrombolysis, *TIA* transient ischaemic attack, *VA*-*ECMO* venoarterial extracorporeal membrane oxygenation

PE was diagnosed with CTPA in 17 patients. In 4 patients presenting with circulatory arrest, a presumptive diagnosis was made using transthoracic echocardiography and later confirmed with pulmonary angiography. Nine patients with high-risk PE underwent thrombosuction because of ST treatment failure. Following ST, these patients all required high-dose vasopressors and 6 required VA-ECMO. Fifteen patients underwent thrombosuction because of absolute contraindications to ST. Of these, 4 had recently undergone major surgery (colectomy, knee surgery, left ventricular assist device placement, pulmonary lobectomy). Others presented with simultaneous intracranial bleeding, concomitant ischaemic stroke with risk of haemorrhagic transformation, clopidogrel use for recent ischaemic stroke, haemophagocytic lymphohistiocytosis with bleeding diathesis, recent trauma and pregnancy with recent chest tube implantation, respectively.

**Fig. 3:**
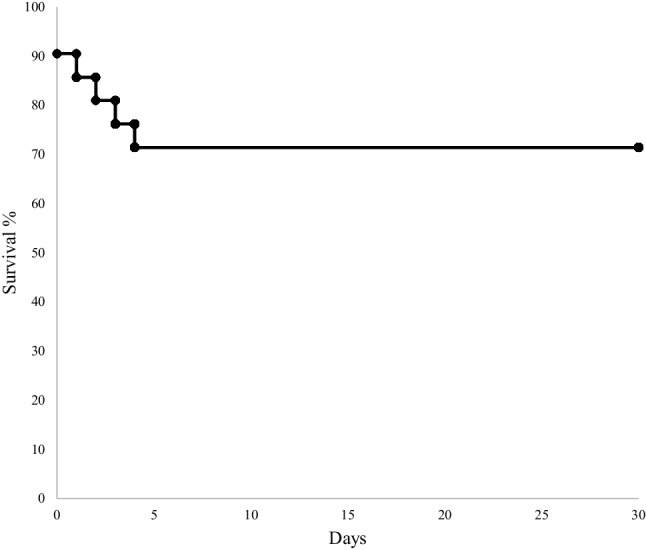
Survival at 30 days following use of the FlowTriever system

The technical success rate was 100%. Six patients (29%) died during 30-day follow-up (Fig. 3), 4 of whom presented with ongoing circulatory arrest (Tab. 2).

**Table 2:** Characteristics of patients who died during admission

Age	Sex	Presentation	Died (days after thrombosuction)	Description
37	Female	Arrest	2	Presentation with circulatory arrest; ST was given and VA-ECMO was initiated; active treatment discontinued after poor neurological status following extensive resuscitation
82	Female	Arrest	1	Presentation with circulatory arrest; ST was administered pre-hospital; shortly thereafter cardiac tamponade developed with obstructive shock; active treatment was stopped due to poor prognosis
37	Male	Shock	0	Presentation with haemophagocytic lymphohistiocytosis complicated by multiorgan failure and invasive pulmonary fungal disease; PE and superior vena cava syndrome developed; deterioration during the FlowTriever procedure after which the patient died due to obstructive shock
56	Female	Arrest	4	Presentation with circulatory arrest; ST was initiated; 3 days later a CT scan showed cerebral ischaemia with oedema and herniation; active treatment discontinued after poor neurological status
50	Male	Arrest	0	Presentation with circulatory arrest; deterioration on ICU after the FlowTriever procedure with refractory respiratory failure
54	Male	Shock	3	Presentation with haemodynamic instability following circulatory arrest in referring centre; ST given in the referring centre; active treatment discontinued after poor neurological status following extensive resuscitation

*DVT* deep venous thrombosis, *ICU* intensive care unit, *PE* pulmonary embolism, *ST* systemic thrombolysis, *VA-ECMO* veno-arterial extracorporeal membrane oxygenation

A major bleeding was recorded in 5 patients (24%) (Tab. 3). In 4 of these patients systemic thrombolysis was administered. In 1 patient the major bleeding event (cardiac tamponade) was considered to be related to the FlowTriever procedure. This patient had undergone a coronary angiogram (before the PE diagnosis was made), received a loading dose of ST and had briefly been resuscitated.

**Table 3:** Major bleeding following the FlowTriever procedure

Age	Sex	ST	Description
37	Female	Yes	Continuous bleeding along VA-ECMO cannula on day 1, requiring administration of 6 packed red blood cells
82	Female	Yes	Presentation with circulatory arrest, followed by coronary angiogram before pulmonary embolism diagnosis. Cardiac tamponade on the same day, after which treatment was ceased due to poor prognosis
56	Male	No	Haemothorax and splenic bleeding following cardiac resuscitation requiring thoracic drain insertion and splenectomy
56	Female	Yes	Haemothorax caused by possible bleeding of intercostal artery on day 1 for which coiling was considered (but not attempted as no active bleeding seen on angiography) and 2 packed red blood cells were administered
56	Male	Yes	Pectoral muscle bleeding following resuscitation; multiple packed cells were administered

*ST* systemic thrombolysis*, VA-ECMO* veno-arterial extracorporeal membrane oxygenation

Of the 15 patients admitted to and discharged from the Intensive Care Unit (ICU), the median time to discharge was 3.5 days (1.3–9.3). Six patients (29%) required VA-ECMO. Of these patients, 2 died with VA-ECMO in situ. The median time to successful weaning of the 4 surviving patients was 3 days (2.3–3.8).

### RV response to thrombosuction

**Fig. 4:**
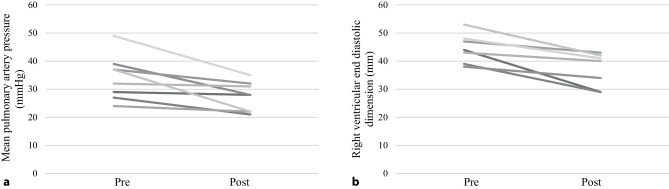
**a** change in mean pulmonary artery pressure following FlowTriever, **b** change in right ventricular end-diastolic (basal) dimension

All 8 patients in whom mPAP was invasively recorded, a reduction was seen in mPAP (median 35 mm Hg (28–39) before versus 28 mm Hg (22–32) after, *p* = 0.012) (Fig. 4a). In 7 patients RV dimensions were recorded, as shown in Fig. 4b. The median RV dimension before the FlowTriever procedure was 44 mm (39–48) versus 40 mm (29–42) after, (*p* = 0.018).

### Pregnancy

In this cohort, a remarkable recovery was seen in two young pregnant patients, successfully treated with thrombosuction. The first patient was a 30-year old female, 29 weeks pregnant, initially hospitalised with a circulatory arrest attributed to hypoxaemia triggered by an epileptic seizure. Chest compressions were complicated by a pneumothorax, for which a chest drain was placed. Later during admission, she developed high-risk PE. Pregnancy and the chest drain were regarded as absolute contraindications for ST. Thrombosuction retrieved a large thrombus load. Shortly thereafter clinical improvement was seen, mainly reflected by a significant reduction in oxygen demand (from 10 litres through a venturi mask to 5 litres through a nasal cannula). A successful caesarean section was performed at 34 weeks of pregnancy.

The second patient was a 23-year-old female, 9 weeks pregnant, presenting with a circulatory arrest due to PE. VA-ECMO was initiated out of hospital by the Helicopter Emergency Medical Services. She had a history of pulmonary embolism during a previous pregnancy but was no longer on anticoagulation. Thrombosuction was initiated to ensure a rapid reduction in thrombus load and allow quick VA-ECMO weaning. A significant amount of thrombus was aspirated with bilateral restoration of flow. Due to persisting respiratory insufficiency, an upgrade was made to veno-arterial-venous-ECMO (VAV-ECMO). Two days later the VAV-ECMO was successfully weaned and removed without the need for inotropic support. On echocardiography, RV dimensions normalised after 10 days. Unfortunately curettage of the foetus had to be performed at admission. Both patients recovered without neurological complications.

## Discussion

Here we describe, for the first time, the use of the FlowTriever system with large-bore percutaneous thrombus aspiration for intermediate-high and high-risk PE in the Netherlands. Using the FlowTriever system, the technical success rate was 100%, 30-day all-cause mortality was 29% and major bleeding was recorded in 24% of patients, of which 1 bleeding complication seemed to be directly related to the FlowTriever procedure. Furthermore, a significant reduction was seen in mPAP and RVEDD.

Treatment of massive PE using thrombolysis remains a challenge in daily practice due to the high mortality and bleeding rates, particularly in high-risk patient subgroups [6]. In current guidelines, ST is recommended in high-risk PE in order to reduce RV failure and mortality [4]. Unfortunately, evidence supporting these recommendations is slim [13, 14]. Importantly, ST is known to be associated with a concomitant high risk of bleeding and may therefore not be suitable in a select group of patients. A meta-analysis reported on 12 studies comparing ST with anticoagulation [7]. Mortality in high-risk and intermediate-risk PE patients receiving ST was 6.9 and 1.0%, respectively. Major bleeding in all patients treated with ST was 9.9% (1.7% fatal or intracranial haemorrhage). The largest trial in this meta-analysis (PEITHO) included 1005 intermediate-risk patients, randomised to ST plus heparin versus placebo plus heparin [14]. All-cause mortality in the ST group was 1.2% and major bleeding 11.5%. The study protocol, however, dictated the exclusion of patients with a significant bleeding risk or any other condition which the investigators felt would cause harm.

Studies including intermediate-risk patients treated with the FlowTriever system initially showed low mortality and bleeding rates. In 2019 the FLARE study enrolled 106 intermediate-risk patients treated with the FlowTriever system of which only two were concomitantly treated with ST [15]. One death was reported and one major bleeding. Similar results were reported by Wible et al. in their study on the use of the FlowTriever system in 46 intermediate-high and intermediate-low-risk patients [11]. In-hospital mortality was 0% and major bleeding was seen in 4.6%. In a small case series with 8 patients (1 high risk, 7 intermediate risk) a mortality rate of 13% was seen, without any bleeding complications [10]. More recently, Luedemann et al. reported on the use of the FlowTriever system in a cohort of intermediate-high and high-risk patients, with a 19% all-cause 30-day mortality and 19% major bleeding [16]. A study by Kucher et al. enrolled 15 high-risk PE patients with a contraindication for ST and reported one death [17]. Eight of these patients received VA-ECMO. Recently, the FLAME trial enrolled 53 high-risk patients [18]. More than one-fifth of these patients presented after circulatory arrest. Major bleeding was seen in 11% and mortality was as low as 1.9%, although notably comatose patients and patients who had been resuscitated for more than 30 min were excluded. Lastly, the ongoing FLASH registry investigators reported on their findings in 800 intermediate-risk and high-risk (8%) patients [19]. In 32% ST was contraindicated and ST had failed in 10%. All-cause 30-day mortality was low (0.8%) and major bleeding at 48 h was 1.4%.

Mortality rates were lower in these studies compared with our results. However, this can be attributed to the significant proportion of patients in our study presenting with high-risk PE (67%) and circulatory arrest (48%). Circulatory arrest due to PE has a detrimental outcome with a high mortality rate of up to 90% [20] and in our study 4 out of 6 deceased patients presented with arrest.

Major bleeding rates in our study were relatively high. Again, this is most likely a reflection of our indiscriminate patient selection where 8 patients (38%) with ST failure and 13 patients (62%) with a major bleeding risk factor were included. Four out of five patients with a major bleeding had received ST prior to the FlowTriever procedure. Importantly, only one major bleed seemed to be related to the FlowTriever procedure (cardiac tamponade).

In our centre, the FlowTriever procedure is performed by a dedicated interventional cardiology team that is proficient in large-bore access and has experience in RV (outflow tract)/pulmonary artery interventions. Furthermore, the on-site availability of (rescue) VA-ECMO offers additional salvage options in patients presenting with circulatory arrest or obstructive shock; the use of VA-ECMO can reduce mortality rates to 7–35% based on previous literature [17, 20–22]. VA-ECMO is initiated in the Cath Lab, using an ultrasound and fluoroscopy assisted protocol, which allows swift concomitant FlowTriever use with minimal delay. In this setting, mechanical thrombectomy can result in a rapid return of circulation due to clot removal, thereby reducing the duration of RV failure and hypoperfusion.

### Limitations

Our study has several limitations. The relatively modest sample size, single-arm design and heterogeneity of the cohort pose challenges in drawing definitive conclusions. Furthermore, the availability of comprehensive haemodynamic, echocardiographic and procedural data varied among patients and was limited, especially in those patients presenting in the most severe clinical condition, i.e. circulatory arrest. Consequently, it is important to recognise that the positive effect on mPAP and RV dimensions may be overestimated and conclusions must be drawn with caution. Thirdly, haemodynamic parameters such as heart rate and blood pressure may have been influenced by inotropics and/or vasopressin and may therefore not correctly reflect the true clinical condition. Finally, two patients were lost to follow-up as they were transferred back to the referring centre within a few days. Procedure-related complications were not seen; however, bleeding complications within 30 days may have been missed. Survival status was available for all patients.

Future questions to be addressed include whether mechanical thrombectomy alone is superior to ST when testing for mortality safety outcomes, and overall cost. Furthermore, which patients are ideal candidates for this procedure is yet to be determined. Studies that are currently ongoing could shed light on this matter. The PEERLESS study will compare mechanical thrombectomy with catheter-directed thrombolysis [23]. Simultaneously, the PEERLESS II trial is a randomised controlled trial currently recruiting (NCT06055920) and will compare mechanical thrombectomy with anticoagulation versus anticoagulation alone in intermediate-risk patients. Furthermore, the investigators of the ongoing FLASH registry, a large multicentre study on the use of mechanical thrombectomy in intermediate-high and high-risk PE, will continue to report on their findings in the future. Finally, the TORPEDO-NL trial is currently recruiting and will randomise high-risk PE patients to mechanical thrombectomy or ST.

## Conclusion

In high-risk patients and in selected intermediate-high-risk patients, when ST is contraindicated or has failed, mechanical thrombectomy using the FlowTriever system seems to be a relatively safe treatment option with often direct haemodynamic improvement and low procedural bleeding risk. Future prospective studies in larger patient populations are needed to reveal which patient group could benefit most of this technique.

## Supplementary Information

Patient en procedural characteristics
