# Supplementary material for: Catheter-directed therapy with the FlowTriever system for intermediate-high and high-risk pulmonary embolism: a single-centre experience
Source: Neth Heart J. 2024 Dec 10;33(1):14–25. doi: 10.1007/s12471-024-01916-1 (PMC11695530; doi:10.1007/s12471-024-01916-1)
Supplement: Supplementary file 1 — Patient en procedural characteristics [file 12471_2024_1916_MOESM1_ESM.docx]

Supplemental Table

| Age, Sex | Risk stratification | Cardiac arrest | ST | Mechanical ventilation | VA-ECMO | Major bleeding | Deceased |  |
| --- | --- | --- | --- | --- | --- | --- | --- | --- |
|  |  |  |  |  |  |  |  |  |
| 71, F | Intermediate-High | - | Contraindicated | - | - | - | - |  |
| 69, F | Intermediate-High | - | Contraindicated | - | - | - | - |  |
| 59, F | Intermediate-High | - | Contraindicated | Yes | - | - | - |  |
| 45, F | Intermediate-High | - | Contraindicated | - | - | - | - |  |
| 37, M | Intermediate-Low | - | Contraindicated | Yes | - | - | Yes |  |
| 57, F | Intermediate-High | - | Contraindicated | - | - | - | - |  |
| 30, F | Intermediate-High | - | Contraindicated | - | - | - | - |  |
| 54, M | High | - | Contraindicated | Yes | - | - | Yes |  |
| 56, M | High | Yes | Contraindicated | Yes | Yes | Yes | - |  |
| 50, M | High | Yes | Failure | Yes | Yes | - | Yes |  |
| 68, F | High | - | Contraindicated | - | - | - | - |  |
| 23, F | High | Yes | Failure | Yes | Yes | - | - |  |
| 68, M | High | Yes | Failure | Yes | Yes | - | - |  |
| 61, M | High | Yes | Contraindicated | Yes | - | - | - |  |
| 37, F | High | Yes | Failure | Yes | Yes | Yes | Yes |  |
| 82, F | High | Yes | Failure | Yes | - | Yes | Yes |  |
| 63, F | High | - | Contraindicated | - | - | - | - |  |
| 63, M | High | Yes | Failure | Yes | - | - | - |  |
| 38, M | High | - | Contraindicated | Yes | - | - | - |  |
| 54, F | High | Yes | Failure | Yes | Yes | - | - |  |
| 56, F | High | Yes | Failure | Yes | - | Yes | Yes |  |

*ST,* systemic thrombolysis, *VA-ECMO*, extracorporeal membrane oxygenation, *F,* female, *M,* male

Table Procedural characteristics and complications

| **Procedure** | |
| --- | --- |
| Fluoroscopy time, minutes | 27 (17-32) |
| Radiation dose, mGy | 101 (47-129) |
| Contrast dose n=9, mL | 150 (100-225) |
| CPR during procedure (patients) | 10 |
| VA-ECMO backup during procedure (patients) | 6 |
| **Device related complications** | |
| Bleeding | 0 |
| Tamponade | 1 |
| Perforation | 0 |
| Infection | 0 |

Data are displayed as median (interquartile range)

*CPR,* Cardiopulmonary resuscitation, *VA*-*ECMO,* Veno-arterial extracorporeal membrane oxygenation
